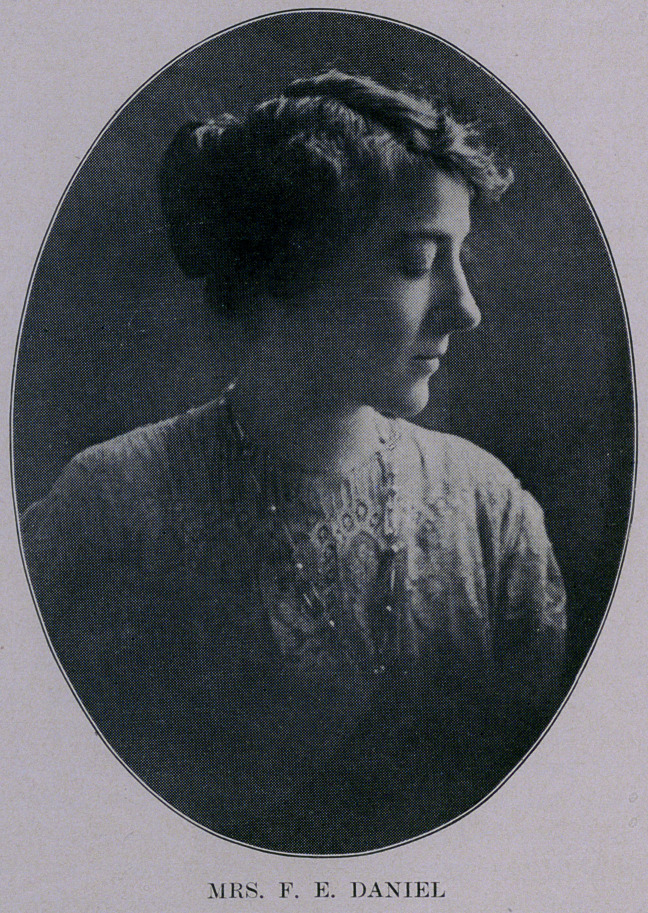# Editorial Department

**Published:** 1913-05

**Authors:** 


MRS. F. E. DANIEL

### EDITORIAL DEPARTMENT.

**DR. F. E. DANIEL, Editor** **DR. R. H. L. BIBB, Associate Editor**

DEPARTMENTS { EUGENICS: Drs. M. Duggan and T. Y. Hull.

WOMAN'S DEPARTMENT: Mrs. F. E. Daniel.

---

THIS issue of the JOURNAL being delayed by press of State printing, I attended the annual convention of the State Medical Association at San Antonio May 6, 7 and 8. I do not propose to report the meeting—the association has a journal to do that—but I jot down a few items for news to my readers.

There were over 700 registered. Lots of women in attendance—wives and daughters of members. Houston gets the 1914 convention. Dr. Marvin L. Graves, of Galveston, professor of medicine in the Medical Department of the University of Texas and chairman of the faculty, was unanimously elected president. Dr. Holman Taylor, secretary of the association and editor of the association's journal, was unanimously and uproariously and enthusiastically re-elected, and, I believe, there was some throwing up of hats. He has made good, with a capital G. Dr. W. A. King of San Antonio was re-elected councilor of the Fifth District, and all the councilors whose time was out were re-elected except in the Third District Dr. C. W. Dickey of Memphis, Texas, defeated Dr. H. D. Barnes of Childress. The vice-presidents are taken from the district society presidents, and Drs. J. H. Foster of Houston, J. C. Anderson of Plainview and R. R. White of Temple were chosen. Dr. A. C. Smith, the long-time treasurer, declined re-election, and Dr. W. L. Allison of Fort Worth was elected. Dr. C. E. Cantrell, member of the board of trustees of the State association journal, was sick and absent. He was much missed. His time as trustee was out, and the retiring president, Dr. J. S. Turner, was elected to succeed him. The delegates to the A. M. A. are Drs. C. A. Smith, Texarkana; Clay Johnson, Fort Worth, and the irrepressible Holman Taylor. Alternates are Drs. F. Paschal, San Antonio; Joe Becton, Greenville; O. L. Norsworthy of Houston.

I haven't room for any more, but I am glad to beat the other fellows to it with this much.

---

IN THE gastro-intestinal derangement of young children, as well as in anemia, loss of appetite, and to promote assimilation when the appetite is impaired, Elixir Maltopepsine (Tilden's), will be found of the highest efficiency.

## Figures and Tables

**Figure f1:**